# An Experimental Anodized Titanium Surface for Transgingival Dental Implant Elements—Preliminary Report

**DOI:** 10.3390/jfb14010034

**Published:** 2023-01-06

**Authors:** Jakub Hadzik, Paweł Kubasiewicz-Ross, Tomasz Gębarowski, Natalia Waloszczyk, Artur Maciej, Agnieszka Stolarczyk, Tomasz Gedrange, Marzena Dominiak, Ernest Szajna, Wojciech Simka

**Affiliations:** 1 Department of Dental Surgery, Faculty of Medicine and Dentistry, Medical University of Wroclaw, 50-425 Wroclaw, Poland; 2 Department of Biostructure and Animal Physiology, Wroclaw University of Environmental and Life Sciences, 51-631 Wroclaw, Poland; 3 Faculty of Chemistry, Silesian University of Technology, 44-100 Gliwice, Poland; 4 Department of Orthodontics, TU Dresden, 01069 Dresden, Germany; 5 WEA Techlab sp. z o.o., 41-301 Dąbrowa Górnicza, Poland

**Keywords:** implant abutment, anodic oxidation, corrosion resistance, implant surface, dental implant

## Abstract

The characteristics such as microtopography, physical and chemical properties influence the behavior of an implant in a soft tissue. Anodization—as a potent method of titanium alloy surface modification—of the transgingival abutment or healing screw, has achieved some improvement. One of the possible surface treatment method is low-pressure radiofrequency oxygen plasma treatment. The aim of the study was to evaluate the chemical properties and cytocompatibility of the experimental surface. Titanium discs made of grade-23 titanium alloy (Ti-6Al-4V) anodized (A sample) with different voltage parameters (28, 67, 78, and 98 V) were included in the study. Half of the samples regarded as the “S” group were additionally treated with low-pressure radiofrequency oxygen plasma treatment. The surfaces were characterized using scanning electron microscopy, X-ray spectroscopy and Raman spectroscopy, and electrochemically investigated via a corrosion test. Furthermore, two cell lines were used, including the CHO-compatible reference line and a primary human fibroblast line for the MTT assay; direct (contact) cytotoxicity of the materials was tested with the cells, and the growth of fibroblasts on the surfaces of the different materials was tested. The morphology of the “S”-treated samples did not differ from the morphology of only-anodized samples. However, the oxygen concentration on the surface in that group slightly increased by about 1% as a result of post-trial treatment. The highest corrosion resistance was observed for both A-78 V and S-78 V samples. The cytotoxicity assay revealed no changes in cell morphology or vitality. The MTT test proved comparable culture viability among all groups; however, the “S” samples showed statistically significantly higher fibroblast proliferation and adhesion scores compared to the “A” samples. Through the in vitro study, the low-pressure radiofrequency oxygen plasma treatment of the anodized Ti-6Al-4V alloy presented itself as an auspicious option in the field of transgingival element surface modification of implants.

## 1. Introduction

Titanium (Ti) dominates contemporary dental implantology, mostly due to its excellent material properties and biological activity [1]. The prevalence of clinical dental implant-related complications is even increasing nowadays with the increasing popularity of dental implant treatment. One of the most current of these is peri-implant mucositis, having a prevalence from 5% to 48% in all dental implant sites in 9–14 years of follow-up [2,3]. The transgingival part of the implant, when exposed to the oral microflora, can be invaded by bacteria, including periopathogenes [4,5].

The long-term predictability of the effectiveness of implant treatment is based, among others, on the creation of a soft-tissue barrier. The biological seal of the soft-tissue–implant interface is created by the epithelium and connective tissue and isolates the implant and the bone surrounding the implant from the oral environment by attaching epithelial and connective tissue [6]. This reduces the infiltration of oral biofilm bacteria and consequently limits the risks of peri-implant mucositis and bone loss. 

The reason for the higher prevalence of peri-implant mucositis when compared to, e.g., gingivitis, lies in, among others, the microscopic differences between peri-implant soft tissue and tissue at the dento-gingival junction. One of them is the pattern of collagen fibers and fibroblasts distribution. Differences in microscopic featuring, and especially in fibroblast distribution, seem to influence the strength of the peri-implant soft tissue junction and result, among other things, in a different biological width of the soft tissue surrounding the implant [7].

Over the years, specific surface properties, such as microtopography, have been investigated to help enhance the soft-tissue attachment and to prevent the formation of bacterial biofilms. Previously used smooth abutments, due to their lesser ability to form plaques, were eventually replaced with those with a rougher surface. The reason for this was some histological investigations in humans and animals that support the fact that moderately rough surfaces can favor soft-tissue integration [8,9].

Titanium dioxide comes in three polymorphs: anatase, rutile, and brookite; the biological activity of TiO_2_ polymorphs is different. The most widely used are anatase and rutile, of which anatase is more chemically reactive. Anodic oxidation (anodization) and thermal treatment demonstrated the ability to form a crystalline TiO_2_ layer on a pure or alloyed titanium surface [10]. Although the possibility of further advancement in implant surface microtopography is believed to reach its limit, many efforts are nowadays devoted to the scope of chemical and electrochemical properties of the surface. These include corrosion resistance, surface wettability, thickness of a titanium dioxide (TiO_2_) layer, and ion adhesion. More precisely, hydrophilicity may promote cell adhesion, which is beneficial during the early stage of wound healing [11]. A negative electrochemical charge, in addition to increasing surface wettability, promotes differentiation and adhesion of mesenchymal stem cells (MSC), and the TiO_2_ layer reduces the microleakage of Ti and its alloys and plays a role in cell adhesion and antibacterial properties. 

Plasma is a versatile tool for the surface modification of metals. Plasmas can be classified as either thermal (hot) plasmas or cold (low pressure) plasmas (CPsor LPPs) [12,13]. Plasma treatment can significantly alter the physicochemical properties of metal surfaces, such as surface chemistry, roughness, wettability, surface charge, and crystallinity, which all play an important role in the biological response of medical materials [14]. Recently, cold plasma has turned out to be a very useful tool for nanotechnology [15].

It is believed that because of this nanoscale modification, positive changes in fibroblasts’ adhesion and viability will be achieved. The result may change the microscopic characteristics of peri-implant junction to better mimic the dental periodontium condition.

The aim of the study was to evaluate the effects of the chemical properties of the novel experimental anode layer and low-pressure radiofrequency (RF) oxygen plasma (OP) treatment of the surface of the transgingival implant element surface on the growth of human fibroblast cells.

The null hypotheses were:

1. None of the experimental samples will express cytotoxicity.

There is no difference between the cytocompatibility of the experimental anodized samples and samples with additional low-pressure radiofrequency oxygen plasma treatment.

## 2. Materials and Methods

For the purpose of this study, titanium alloy samples of different experimental surfaces were delivered and tested. All samples have been characterized in terms of morphology and chemical structure, and electrochemical corrosion investigations were performed. All samples were tested for cytotoxicity and cytocompatibility, and cell lines were used to assess adhesion to each sample.

### 2.1. Titanium Plates’ Preparation and Surface Modification 

For the purposes of the study, NanoPrime (Dębica, Poland) prepared titanium plates made of grade 23 titanium alloy (Ti-6Al-4V) anodized with different voltage parameters. In the study, 8 different surfaces (5 each surface) were used. All samples were discs with a diameter of 5.3 mm and a height of 7.0 mm. The details of the titanium surface samples used in the study are presented in Table 1.

The samples were anodized with the use of an Ametek power supply in a glass electrolyzer. The sample served as an anode, and the cathode was made from stainless steel. Anodizing was performed at constant current density (*j* = 50 mA/cm^2^) up to a limited voltage (see Table 1). For the anodizing process, two types of electrolytes, acidic and basic, were used (Table 1). The anodized samples were cleaned in demineralized water and dried in air. Half of the samples were additionally treated with the use of a low-pressure radiofrequency oxygen plasma (low-pressure RF OP) for “S” samples. For this purpose, the samples were placed in a vacuum chamber for 5 min (frequency 40 MHz; power 500 Watt). During this time, oxygen was pumped (1 L/min).

### 2.2. Morphology and Chemical Characterization of the Anodized Samples

The morphologies and elemental compositions of the anodized samples were investigated with the use of a scanning electron microscope (SEM) equipped with an energy-dispersive X-ray spectrometer (EDX) (accelerating voltage—15 kV; Phenom ProX (Thermo Fisher Scientific Inc., Waltham, MA, USA). Raman spectroscopy was used for determination of chemical compounds on the investigated surfaces. Experimental samples of Raman spectra were recorded using a Raman microscope (inVia Renishaw, Gloucestershire, UK) equipped with a charge-coupled device (CCD) detector, using a green (514 nm) laser light source. Calibration was performed with a silicon (Si) calibration sample prior to measurements, which were carried out using an Olympus LMPlanFl 50× magnification lens (Olympus Corporation, Tokyo, Japan), with a 5 s acquisition time in the 100–3000 cm^−1^ range and 10 accumulations per spectrum, for at least 5 randomly selected points on the cylindrical surfaces of the samples. 

### 2.3. Electrochemical Corrosion Investigations

The rifled samples of anodized samples with a diameter of 7.0 mm and a height of 5.3 mm were used for the corrosion studies. Additionally, for comparison purposes, corrosion research was also conducted for a nonoxidized titanium sample, which was the reference material.

Corrosion studies were carried out according to the ISO 10933-15 standard, entitled: “Biological evaluation of medical devices—Part 15: Identification and quantification of degradation products from metals and alloys”. The electrochemical research was realized according to the potentiodynamic test, which was used to determine the general electrochemical behavior of the materials and to define their passive limit potential (*Ea*) and breakdown potential (*Ep*).

The tests were carried out in a 150 cm^3^ glass electrolytic cell (according to the ISO 3585 standard) while maintaining the temperature within ±1 °C. The counter-electrode was platinum mesh. The Autolab 100 N potentiostat-galvanostat (Metrohm Autolab, Herisau, Switzerland) was used for the studies. The corrosion medium was a 0.9% NaCl solution (Fresenius Kabi, Poland) with a pH equal to 5.7 ± 0.1. Reduced oxygen level in the solution was achieved by argon purging (100 cm^3^/min; 30 min) before the start of the studies. The blow of argon was continued during the measurements, which ensured mixing of the solution and sustained the lack of oxygen in the medium. The studies were carried out at a temperature of (37 ± 1) °C.

After two hours from the moment of immersion of the working electrode (studied material), the open-circuit potential (*E_OCP_*) was measured, and it was the starting potential for potentiodynamic measurements. The sample was tested by linear sweep polarization with a sweep rate of 10 mV/s. The polarization was changed in the range of potential from *E_OCP_* to *E_OCP_* + 2000 mV, and then the sample was scanned in reverse through the reduction of the potential to the *E_OCP_* value. For every sample, the measurement was continued for 5 cycles, which allowed one to achieve the potential of *E_OCP_* + 2000 mV. The obtained results were recorded in the half-logarithmic form of the *i-E* curves. According to the standard, the breakdown potential (*E_p_*) should be defined from the last cycle; however, none of the tested samples were found to achieve this potential. The ISO 10933 standard also provided the measurement at a potential of *E_p_* + 50 mV, but the lack of achieving the *Ep* value precluded it.

The quality of the tested samples was analyzed by microscopic studies by observations of their surface structures before and after corrosion studies. The analysis was performed by a Phenom ProX (Thermo Fisher Scientific Inc., Waltham, MA, USA) scanning electron microscope. Images obtained at ×1000 magnification were recorded for all samples. 

To determine the degradation products formed during the potentiostatic measurements (at *E_p_* + 50 mV), the ISO 10933 standard also provides a qualitative and quantitative analysis of the electrolyte after the completion of these measurements. As there was no possibility of taking measurements, the analysis of the chemical composition of the corrosion medium was not performed.

### 2.4. Biological Analyses 

#### 2.4.1. Cell Culture 

Two cell types were used in the study: primary gingival fibroblasts; normal, human, adult (HGF) from ATCC (Manassas, VA, USA); and CHO cells purchased from Sigma-Aldrich (The European Collection of Authenticated Cell Cultures—ECACC). Cell cultures were carried out in an incubator at 37 °C, in a 5% CO_2_ atmosphere, at 95% humidity. Cells, after thawing, were cultured for at least 2 weeks prior to testing. During culturing, confluence measurement was performed using a Juli Br microscope (NanoEntek, Seoul, Republic of Korea). Cell cultures were passaged once a week with trypsin/EDTA solution. Cells for the assay were counted using a NucleoCounter^®^ NC-200 automatic cell counter (ChemoMetec A/S, Allerod, Denmark). Cells were cultured in Dulbecco’s modified eagle medium (DMEM) without phenol red or F-12K medium (Kaighn’s modification of Ham’s F-12 medium) supplemented with 10% fetal bovine serum (FBS), antibiotics, and L-glutamine (200 mM). Culture reagents were purchased from Biological Industries (Beit-Haemek, Israel). Detailed methodology for the assessment of cell vitality is presented by Hadzik et al. concerning experimental implant surfaces [16]. 

#### 2.4.2. Preparation of Samples 

The specifics of the study require the sterility of the experimental disc samples. The samples were sterilized in an autoclave (Enbio PRO, Enbio Group AG, Oensingen, Switzerland) with a temperature of 121 °C, a pressure of 2.1 bar, and a cycle time of 30 min. For in vitro cytotoxicity tests, liquid extracts of materials were prepared according to the standard provisions: EN ISO 10993-5: Biological evaluation of medical devices—Part 5: In vitro cytotoxicity testing. The test materials were extracted in sealed, chemically inert, sealed tubes for 24 h at 37 °C in a greenhouse. In studies in which the test material was placed in a culture dish, the test material after sterilization was stored at room temperature for up to one week. 

#### 2.4.3. Vitality Assessment

The study was carried out under standard conditions. Cells were obtained from culture bottles by enzyme digestion (trypsin/EDTA), and the cell suspension obtained was centrifuged (200× *g*, 3 min). The cells were then counted. The density of the cell suspension was 1 × 105 cells/mL. Using a multichannel pipette, cells were dispensed at 100 µL into 96-well plates (1 × 10^4^ cells/well). Before starting the experiment, cells were incubated for 24 h (5% CO_2_, 37 °C, 90% relative humidity). This incubation time ensured cell regeneration, adhesion, and transition to the logarithmic growth phase. After this time, 100 µL of medium containing the appropriate sample extracts, control, or blank only, was added to each well. The test plates were incubated for another 24 h (5% CO_2_, 37 °C, 90% humidity). The culture medium was then removed from the plates and 50 µL of 1 mg/mL MTT solution was added to each well. The discs were incubated for another 2 h at 37 °C. After this time, the MTT solution was removed, and 100 µL of isopropyl alcohol was added to each well. Five samples of each material were used to make the extract. The reference wavelength of 570 nm was used to read absorbance on a MultiscanGo reader (Thermo Fisher Scientific, Waltham, MA, USA). 

#### 2.4.4. Co-Culture of Cells with Materials

**Figure 1:**
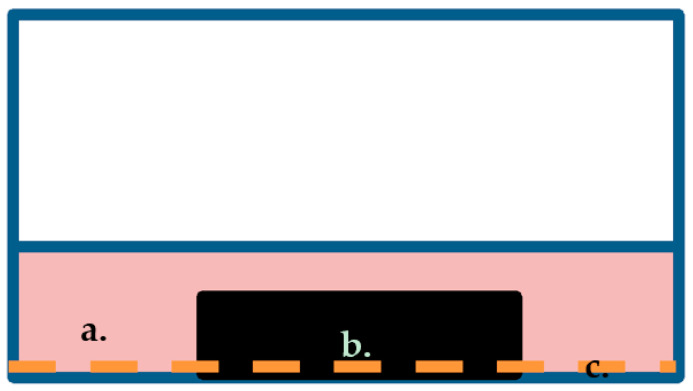
Method of co-culturing of test material with cells: (a) culture medium, (b) test material, (c) cell monolayer.

Cells for this test were prepared in the same way as for the adhesion assessment test. For coculturing, cells were seeded into 24-well plates on which the test materials were placed. In this test, the direct interaction between the cells and the test material was evaluated. Cells were stained with the Cell Viability Imaging Kit (Thermo Fisher Scientific, Waltham, MA, USA) for 15 min at room temperature. Reading was performed using an EVOS FL imaging system (Thermo Fisher Scientific, Waltham, MA, USA). In addition, a cell vitality assessment was also performed according to the methodology outlined above (Figure 1).

#### 2.4.5. Cell Attachment 

Cell lines were also used to study cell adhesion to the tested surfaces. The cell suspension density used for the adhesion test was 1 × 10^6^ cells/mL. Twenty-four well-sized test plates were used for the assay. The test materials were placed in the wells. Cells were applied to the material using an automatic pipette. After application, cells were incubated for 2 h (5% CO_2_, 37 °C, 90% humidity) to allow them to adhere to the test materials. The wells were then supplemented with serum culture medium in a volume of 1000 µL. The test materials with cells were incubated for another 72 h (5% CO_2_, 37 °C, 90% humidity). After incubation time, cells growing on test surfaces were stained using a cell viability ReadyProbes™ Cell Viability Imaging Kit, Blue/Green (Thermo Fisher Scientific, Waltham, MA, USA). Staining was performed by adding the dye to the culture and incubating it for 30 min. Images were then taken using a BioTek Lionheart microscope (Agilent Technologies, Santa Clara, CA, USA) using fluorescence excitation with a led illuminator: ex 377 em 447 and ex 469 em 525. Further analysis was performed using GEN5 dedicated image analysis software (Agilent Technologies, Santa Clara, CA, USA). The fluorescence intensity and number of cells stained with each dye were analyzed. Cells showing blue fluorescence were counted as alive, and green cells as dead.

#### 2.4.6. Assessment of the Induction of Oxidative Stress in Cells (ROS)

Cells were incubated for 24 h with extracts of the test surfaces. CellROX^®^ reagent at a final concentration of 5 μM was then added to the culture, and the cells were incubated for 30 min at 37 °C. After this time, the medium was removed, and the cells were washed 3 times with PBS. The fluorescence of Ex/Em 545/565 nm was then measured. 

### 2.5. Statistical Analysis

All biological tests were performed in five independent replicates. Due to the normal distribution and equal variance of the results obtained, statistical calculations were performed with parametric tests. Using Statistica v.13 software, the statistical significance was calculated using Tukey’s post hoc test using Statistica v.13 software. The significance point was established at * *p* < 0.05. 

## 3. Results

### 3.1. Morphology and Chemical Composition of Anodized Titanium Alloy Samples

**Figure 2:**
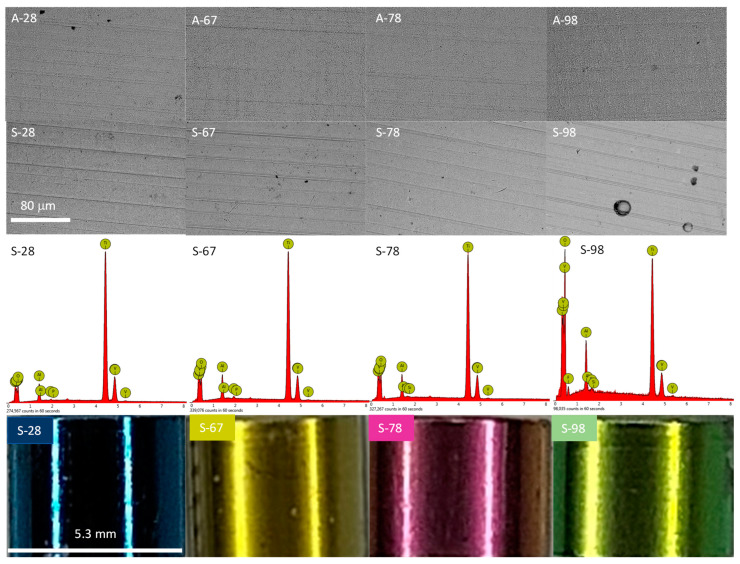
SEM images, EDX spectra, and macro-view of the samples after the anodization process (“A” samples) and after additional oxygen plasma treatment (“S” samples).

The alloy samples were subjected to anodic oxidation using four voltages, 28, 67, 78, and 98 V. Anodizing at 28 and 67 volts was performed in the H_3_PO_4_-based electrolyte, and anodizing at 78 and 98 V was performed in a Na_2_SiO_3_ and NH_4_F-based electrolyte. After that process, oxide coatings of various colors were obtained on the surfaces of the samples (Figure 2). The oxide layers imitated the substrate morphology, practically to a voltage below 98 V, which is typical in the classic anodizing process of titanium and its alloys [17,18]. The morphologies of the samples oxidized at 28, 67, and 78 volts were virtually indistinguishable from each other (Figure 2). The application of higher voltage resulted in the appearance of circular structures, which may have been the effect of adhered oxygen and the beginning of the plasma electrochemical oxidation process (PEO) [19,20].

The anodic oxidation process led to incorporation into the oxide layer formed on the Ti-6Al-4V alloy of the elements present in the electrolyte (Table 2), phosphorus, silicon, and fluorine, respectively. However, the concentrations of these elements were below 0.3 at %, which is also characteristic of classical anodizing. Anodized samples (series A) were additionally treated in an oxygen plasma (series S). The morphology of the “S”-treated samples did not differ from the morphology of the only-anodized samples (Figure 2). It is worth noting that the oxygen concentration on the surface of the samples slightly increased (Table 2, by about 1%. This type of treatment allows one to increase the degree of oxidation of titanium to TiO_2_ [15], which may have a positive effect on enhanced cytocompatibility [21].

**Figure 3:**
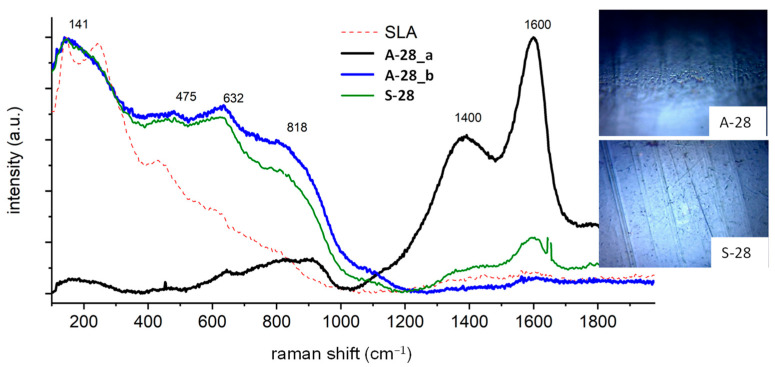
Raman spectra of the A-28 and S-28 samples.

For comparative purposes, in the figures presenting the RM analysis of the obtained samples, the substrate spectrum (SLA) has been drawn with a dashed line. The surface had the same chemical composition, regardless of whether the spectrum was measured at the surface or in the depths of the pores of the sample. The surface composition was a mixture of titanium (IV) oxide in the form of anatase, as evidenced by a very high peak at 144 cm^−1^, and the presence of signals at 250, 444, and 608 cm^−1^ is characteristic of rutile TiO_2_. The spectra also include images taken by optical microscopy, showing the analyzed area of the implants. For sample A-28, the cloud composition of the pores (Figure 3) differs from the sample surface. On the spectrum recorded in the pore A-28_a, there are dominant signals at 1400 and 1600 cm^−1^ originating from carbon-carbon bonds characteristic for the carbonized organic residue occluded inside the pores of sample A-28. The spectrum A-28_b is characteristic of the material surface, which corresponds to the mixture of amorphous titanium oxides in the form of anatase (peak at 144 cm^−1^), aluminum oxide Al_2_O_3_, and vanadium V_2_O_5_; the amorphous nature of the compounds obtained is evidenced by a significant broadening of the signals. Sample S-28, treated with oxygen plasma, had a homogeneous chemical surface, the oxide composition of which was the same as that of sample A-28. There were traces of carbonized organic residue on the entire surface (peaks 1400 and 1600 cm^−1^), the local content of which was lower than that of sample A-28.

**Figure 4:**
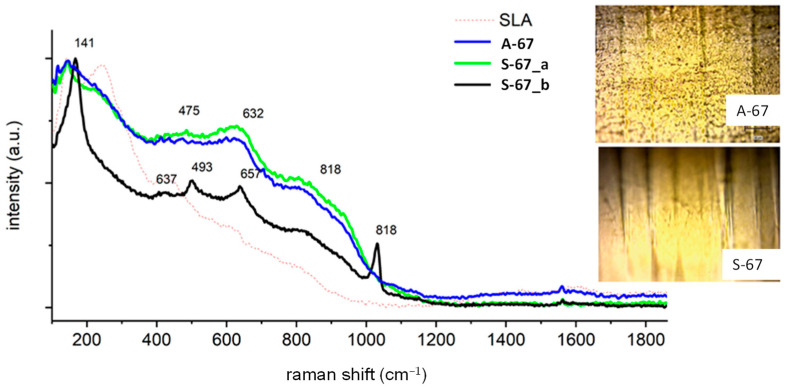
Raman spectra of the A-67 and S-67 samples.

Sample A-67 (Figure 4) was chemically homogeneous, identical in composition to the previous samples. This sample did not contain a carbon trace on the surface. In sample S-67, as in case A-67, no carbon film was observed. However, its surface was not chemically homogeneous, and the heterogeneity itself could not be related to artifacts on the surface. Eighty percent of the sample describes the spectrum S-67_b. The remaining area is covered with well-crystallized anatase and vanadium oxide (signal at 1028 cm^−1^).

**Figure 5:**
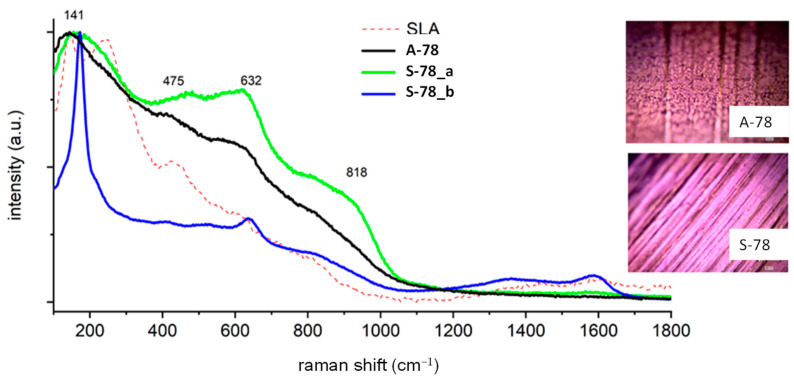
Raman spectra of the A-78 and S-78 samples.

The surface of sample A-78 (Figure 5) was chemically homogeneous, having no carbon traces. It was characterized by the highest degree of amorphousness. Its chemical composition was the same as that of other samples and the most similar to that of sample A-67_b. Sample S-78 (Figure 5) was mainly a mixture of oxides that has already been analyzed. In some areas, however, one could find surfaces covered with crystalline anatase; see Figure 5, S-78_b. As in sample S-67, the presence of the crystal structure could not be associated with surface defects.

**Figure 6:**
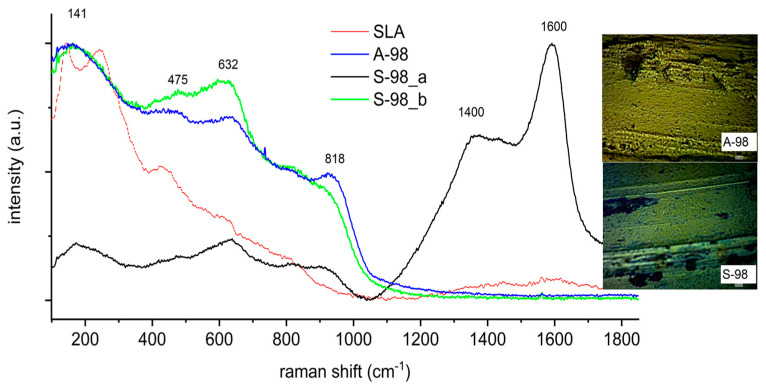
Raman spectra of the A-98 and S-98 samples.

The surface of sample A-98 (Figure 6) was chemically homogeneous; its chemical composition was the same as that of the other samples and the nearest to that of sample A-67_b (Figure 4). The chemical composition of the surface of sample S-98 was similar to that of sample A-28; in this case, areas with strong carbon content in the pores were also recorded. On the surface of the sample, there was mixing of inorganic oxides, with a predominance of titanium oxides.

### 3.2. Electrochemical Corrosion Investigations

The structures of the surface of the anodized titanium samples subjected to the corrosion test and the potentiodynamic curves obtained by the cyclic polarization method are presented in Figure 7, Figure 8, Figure 9, Figure 10, Figure 11, Figure 12, Figure 13, Figure 14, Figure 15, Figure 16, Figure 17, Figure 18, Figure 19, Figure 20, Figure 21 and Figure 22.

Analysis of the results showed that all of the anodized titanium samples tested exhibited very good corrosion resistance under the conditions of examination, and their electrochemical characteristics are similar. The corrosion potential registered after 2 h from the start of the test were in the range from. 107 to 0.134 V and were higher than the *E_OCP_* received for the nonoxidized substrate (−0.212 V; SEM images and potentiodynamic curves not shown). The highest values of the *E_OCP_* values were observed for samples A-78 and S-78 (0.134 and 0.087 V respectively) oxidized in the alkaline bath at the maximum voltage of 78 V, which indicates their high tightness. In none of the cases of the measurements was the breakdown potential achieved; therefore, it was concluded that the formed oxide coatings offer perfect protection for titanium exploited in the chloride environment. It should be added that titanium not subjected to the anodic oxidation process also had good corrosion resistance under those conditions, but it was significantly lower than that of anodized titanium, which results from the course of the curves (the values of the corrosion current density for non-oxidized titanium are about 2–3 orders of magnitude lower). Thus, the results obtained from the studies lets us state that the material may also be also exploited in a chloride environment, even after possible damage to the oxide coating formed on its surface. The polarization of the studied samples up to a potential of 2000 mV higher than the open-circuit potential did not cause significant changes in the structure of the surfaces of the tested samples. This also proves their high corrosion resistance.

### 3.3. In Vitro Cytotoxicity Assessment

**Figure 23:**
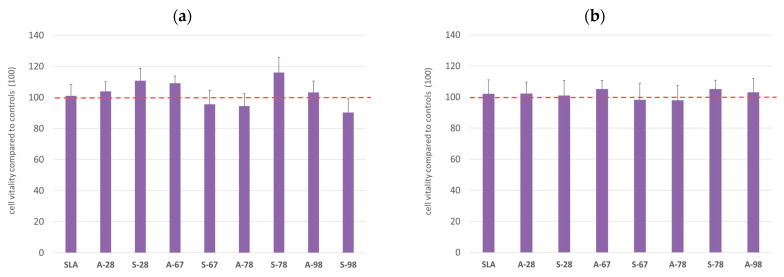
In vitro cytotoxicity testing of materials (HGF cells (**a**) CHO cells (**b**)). The results are averages of five independent experiments, presented as the ratio of the value obtained in the test culture, E, to that of the control, E0 (E/E0). There was no statistically significant decrease in culture viability compared to the control (*p* < 0.05).

The study assessed the materials’ effects on the viability of the two cell lines used in the in vitro cytotoxicity assessment of the materials. For both lines, no toxicity was found for the extracts obtained during the extraction of the biomaterials. The results obtained are shown in the graphs (Figure 23). A CHO-compatible reference line and a primary human fibroblast line were used in the MTT assay (Figure 23a,b). Cell morphology was also assessed for both lines according to the specifications for each line. No changes in cell morphology, the presence of vacuoles, cell membrane fragmentation, or the presence of other cytopathic changes were evident in the cultures.

### 3.4. Co-Culture of Cells with Test Materials

**Figure 24:**
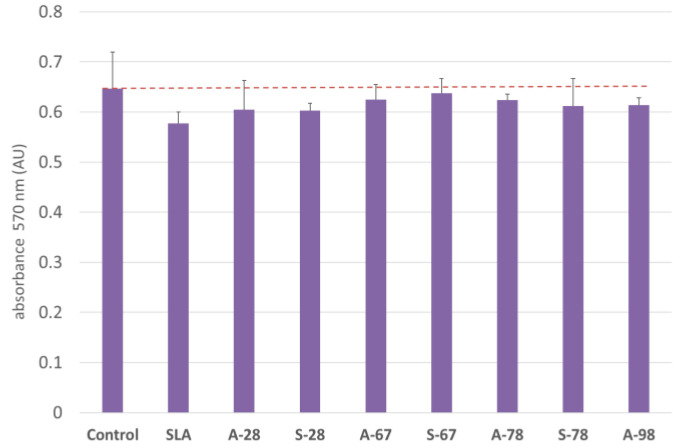
Effect on CHO cell vitality in the MTT assay, for co-cultures with test materials. Results are the averages of five independent experiments. There was no statistically significant decrease in culture viability compared to the control (*p* < 0.05).

**Figure 25:**
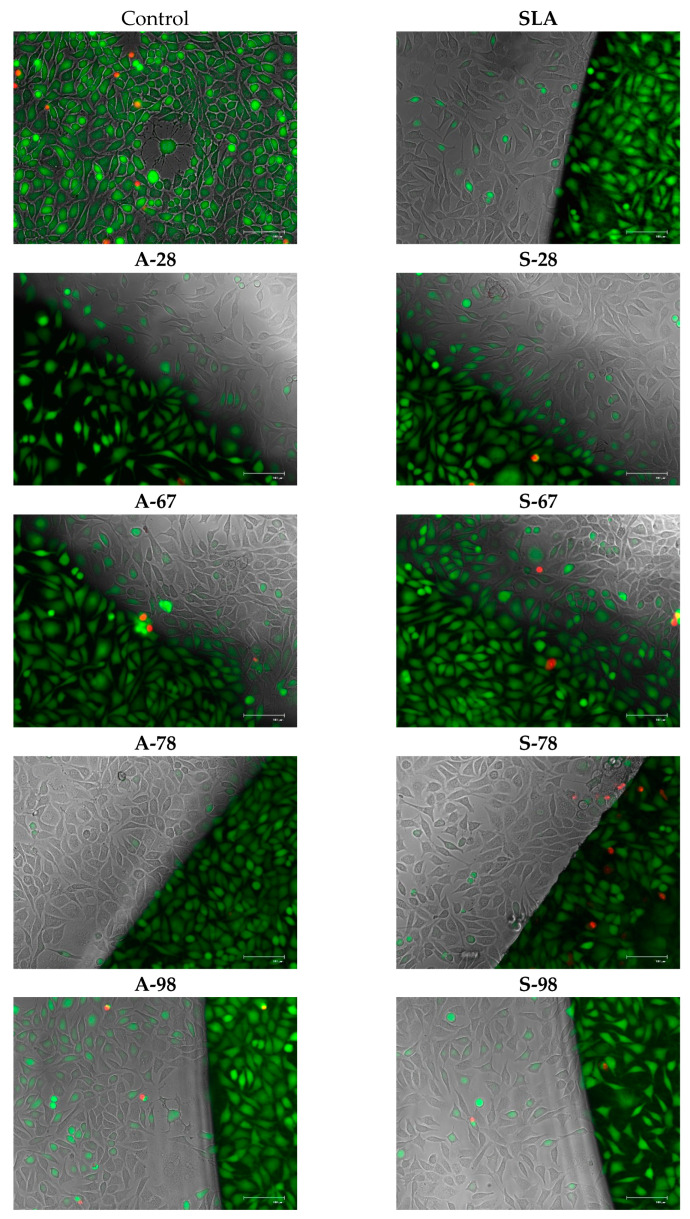
Microphotographs of cells growing together with the test materials. Live–dead staining. Live cells fluoresce green, dead cells fluoresce red. Visible cell growth from near the material and beneath the film. Objective magnification 20×, microscope EVOS FL.

To assess the direct (contact) cytotoxicity of the materials with the cells, co-cultures with the cells were also carried out. During the study, cells were co-cultured with the test materials. For the cultures, an evaluation was performed according to the criteria described previously. The test materials did not show any cytotoxicity. The observations obtained were confirmed by performing viability staining and were also evaluated in the MTT test. The results are presented in Figure 24 and Figure 25.

### 3.5. Adhesion and Cell Growth on the Surfaces of Tested Materials

**Figure 26:**
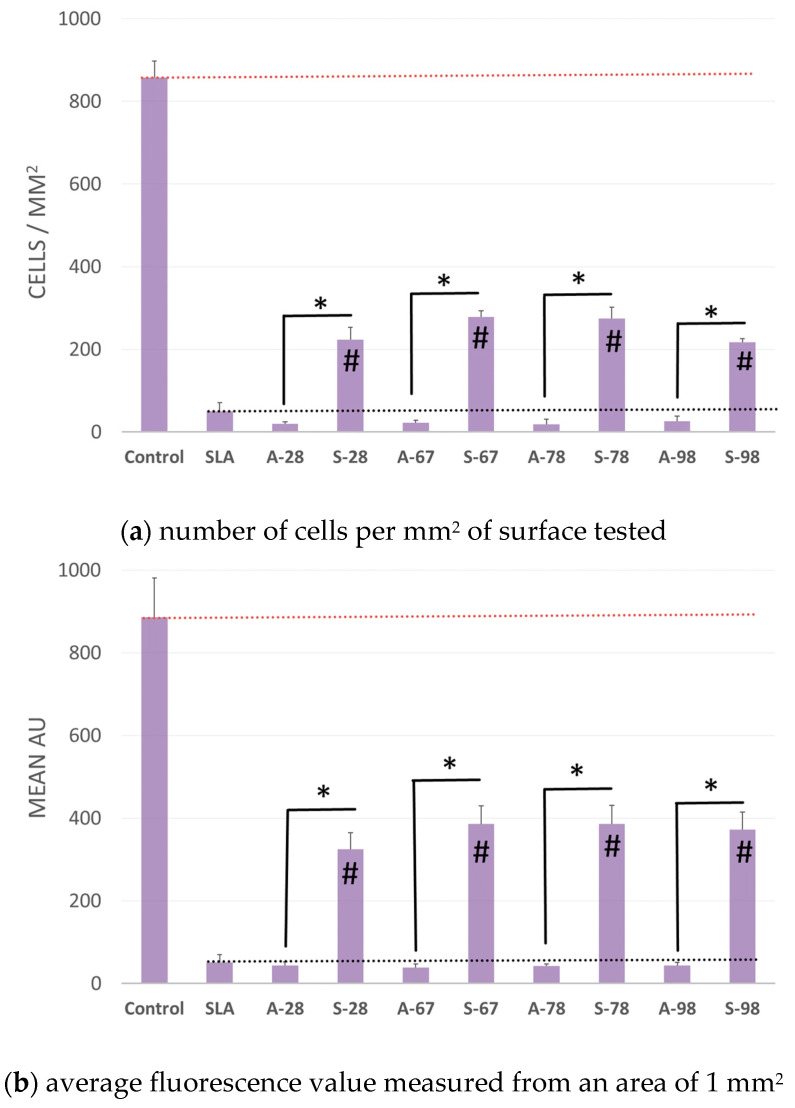
Adhesion and proliferation of human fibroblasts on modified test surfaces after 72 h: (**a**) number of the cells per mm^2^ of test surface, (**b**) mean fluorescence value measured from an area of 1 mm^2^. * Statistically significant difference (*p* < 0.05) compared to control (SLA) and between a and s “#” materials.

**Figure 27:**
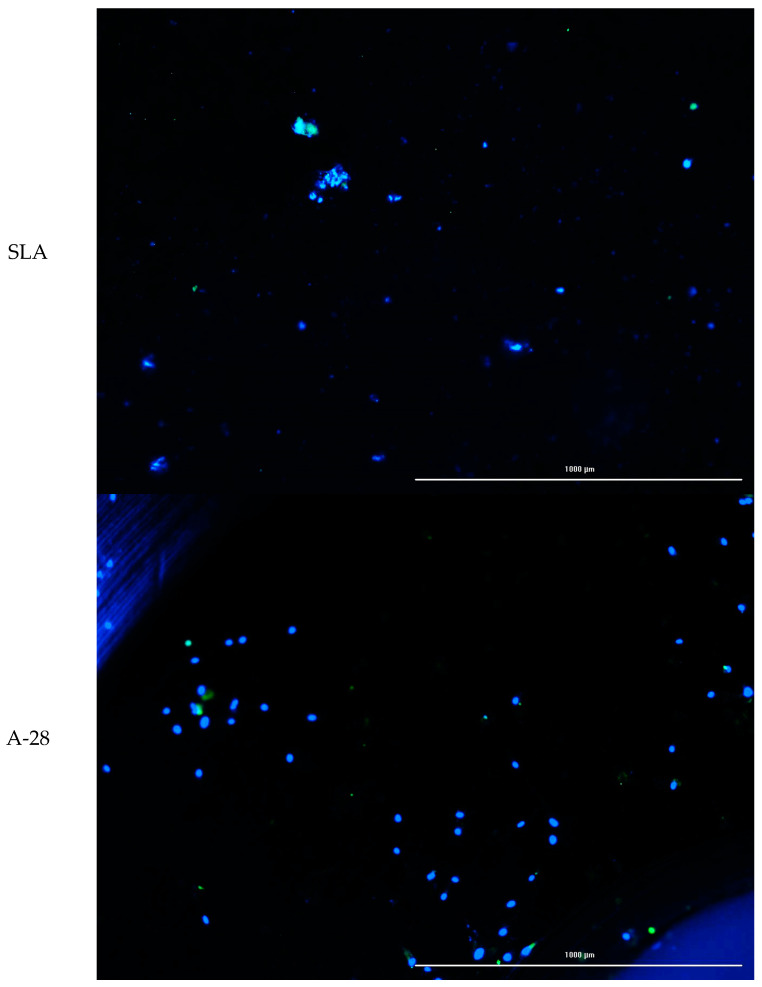

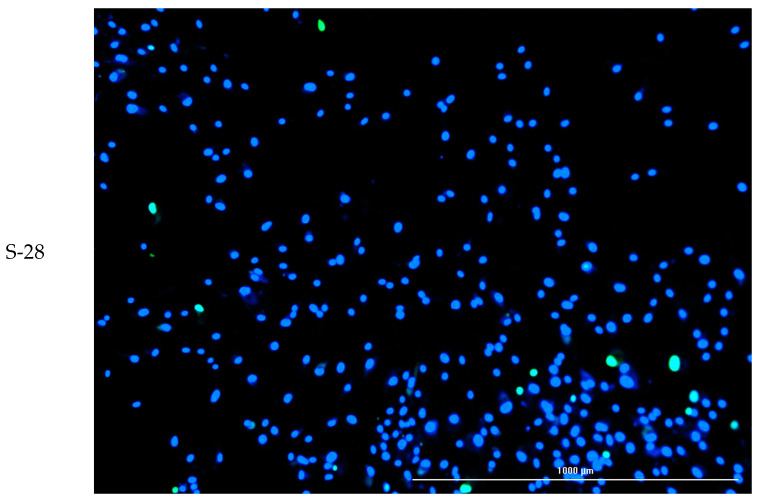
Example of cell growth on the surfaces tested (SLA, A-28, and S-28). Live–dead staining: blue live cells and green dead cells; 10× magnification; EVOS FL microscope.

The aim of the study was to evaluate the growth of normal human fibroblasts on the surfaces of different materials. The results were compared with those of modified polystyrene for cell growth and the reference material SLA. The number of cells with no specific surface area and the average fluorescence value were analyzed. The analysis of the two parameters was due to the possibility of errors in the case of dense cell growth in colony form. The results showed that the A series did not differ from the SLA reference material. For the S series, a reproducible increase in the number of adherent cells growing on the modified surface was obtained. For S-98, adhesion and cell growth improved 4.3 to 5.5-fold for S-67. The increase in fluorescence was greater, by 6.3 to 7.5 times. A comparison of growth on the tested surfaces is shown in Figure 26, and example microphotographs are shown in Figure 27.

**Figure 28:**
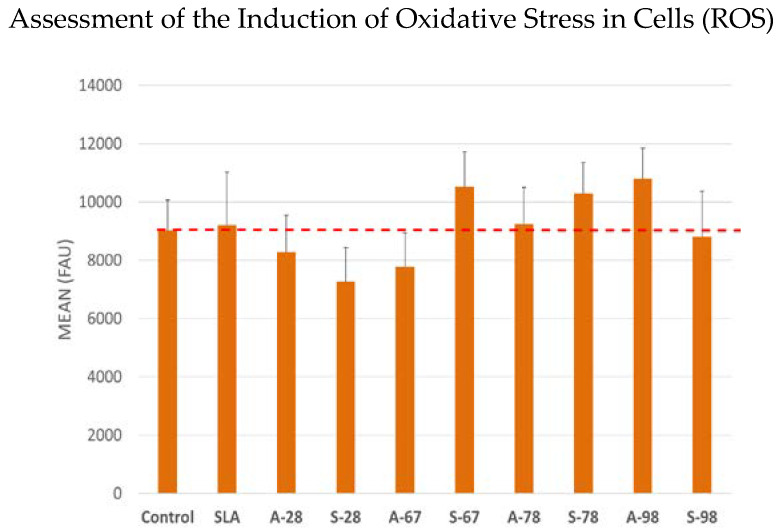
Investigation of oxidative stress in cells incubated with test materials. Results are averages from five independent experiments, presented as the mean fluorescence values read from the test wells. There was no statistically significant effect of the materials on oxidative stress compared to the control (*p* < 0.05).

To confirm safety and exclude negative effects on cells, the effect of test materials on the level of free oxygen radicals (ROS) in cells incubated with the test material was also evaluated. The results showed a neutral effect of the materials on the level of oxidative stress in the cells. The results of the test are presented in Figure 28.

Study research hypotheses:

1. The first research hypothesis was accepted. None of the experimental samples expressed cytotoxicity.
2. The second null hypothesis was rejected. Samples after additional low-pressure radiofrequency oxygen plasma treatment substantially enhanced the cytocompatibility.

## 4. Discussions

An important aim in dental implantology is to make it less traumatic. Therefore, minimally invasive procedures have been introduced to increase patient post-surgical comfort by atraumatic acts [22,23]. Consequently, many new surgical and nonsurgical treatment methods for peri-implantitis management have been described; some demonstrated statistically significant clinical improvements, although the impacts on microbiological and biochemical parameters are still insufficient [24,25,26,27]. Therefore, primary prophylaxis of peri-implant diseases is important and can be achieved with correct treatment planning in terms of implant position, the morphologies of soft tissues surrounding the implant, and the proper prosthetic reconstruction [28,29,30,31].

Moreover, in terms of the possible causation of peri-implant disease, apart from biofilm accumulation, recent studies have focused on whether titanium particles can induce foreign-body reactions due to activation of pro-inflammatory cytokines, resulting in loss of bone and soft tissue loss [32]. Several factors can facilitate implant corrosion in the oral cavity, such as pH, bacteria, chemicals, and other contaminants [33]. The presence of reactive oxygen species (ROS) related to the inflammatory process of the peri-implant tissues can alter the thin passive layer that protects titanium implants against the aggressive environment of the human body [34,35]. Studies in vivo showed titanium degradation as the presence of particles in the periprosthetic areas; various mechanisms were described as causes of titanium release, including corrosion of the implant surface and friction at the implant–abutment interface [36]. Although the implant abutment is constantly in contact with the soft tissue around the dental implant, it is extremely important to choose a surface with the best corrosion-resistant properties. The corrosion resistance and biocompatibility of dental materials are essentially related to the presence of a thin passive oxide layer on the surface [37].

Thus, in our study, in none of the cases of the corrosion test measurements was the breakdown potential reached; therefore, it was concluded that the formed oxide coatings are perfect protection of titanium exploited in a chloride environment. It should be added that titanium not subjected to the anodic oxidation process also exhibits good corrosion resistance, but it is significantly lower than in the case of anodized titanium, which results from the course of the curves (the values of the corrosion current density for non-oxidized titanium are about 2–3 orders of magnitude lower). Therefore, the obtained results of the studies allow us to state that the material may also be used in a chloride environment, even after possible damage to the oxide coating formed on its surface. The polarization of the studied samples up to 2000 mV higher potential than the open-circuit potential did not cause significant changes in the structure of the surface of the tested samples. This also proves their high corrosion resistance.

It is worth noting that the highest values of the *E_OCP_* values were observed in our study for samples A-78 and S-78 (0.134 V, and 0.087 V respectively).

Corvino et al., in the systematic review of in vitro studies, drew attention to the fact that the abutment material and its mechanical, physical, or chemical modifications influence the fibroblast response in vitro, in terms of adhesion, proliferation, cell morphology, and expression of proteins related to the extracellular matrix, especially in the earlier phases of contact with the abutment surface [29]. Anodized abutments and other transgingival elements, such as healing screws, are interesting alternatives to pure titanium abutments because they can be prepared in different colors. Color coding is frequently used for titanium implant healing screws, to ease the fitting of them to particular implant sizes. In our study, the Ti-6Al-4V alloy samples were subjected to anodic oxidation using four voltages—28, 67, 78, and 98 V. Anodizing at 28 and 67 volts was performed in with a H_3_PO_4_-based electrolyte; and anodizing at 78 and 98 V was performed in Na_2_SiO_3_ and NH_4_F-based electrolytes. Color is also important in terms of aesthetics of prosthetic reconstruction; in this case, the coloration concerns the prosthetic abutment. The grayish appearance of titanium abutments, especially when thin, soft tissues are present, can cause shining through the peri-implant soft tissues. Wang et al., in his clinical study, found a gold-anodized and pink-anodized titanium abutment better in terms of soft-tissue esthetics than the non-anodized titanium abutment [38]. Farrag and Khamis [39], in a clinical study comparing anodized and non-anodized healing abutments, have found that anodized titanium abutment collars do not produce a clinically significant effect on the health or esthetics of peri-implant soft tissues compared to non-anodized ones. However, regarding the coloration of the anodized abutment—not only for the aesthetics—this technology when applied also creates different surface characteristics that can modify the tissue integration. Anodization can take titanium through a spectrum of colors, and by increasing the thickness of the oxide layer, changes the abutment surface characteristics. Musssano et al. [40] found that anodized surfaces of dental implant abutments could improve the adhesion of the two major cell types within peri-implant soft tissues, which makes anodized surfaces a promising option for implant dentistry.

Many in vitro studies have used HGF to determine the effects of different surface treatments on cell adhesion and spread to predict the epithelial attachment potential and soft-tissue sealing in the clinical situation [41,42,43]. Our study has confirmed that none of the surfaces tested here have any cytotoxic effect on HGF cell lines, so they can be safe to use for dental implant transgingival elements such as abutments or healing screws. This was confirmed by performing viability staining and an MTT assay, which identified no significant differences between the surfaces. The adhesion and growth test showed that the A-series samples did not differ from the SLA reference material; however, in the S-series samples, a reproducible 3 to 4-fold increase was obtained in the number of cells that adhesively grew on the modified surface. The reason for the significantly increased cell coverage area after plasma treatment could have been the chemical changes in the surface of the “S” sample. 

The anodic anodic oxidation process led to the incorporation into the oxide layer formed on the Ti6-Al-4V alloy of the elements present in the electrolyte: phosphorus, silicon, and fluorine. However, the concentrations of these elements were below 0.3 at %, which is also characteristic of classical anodizing. 

In our study, Ti6-Al-4V alloy represented by the “S” samples were additionally subjected to low-pressure radiofrequency oxygen plasma treatment. The results of EDX analysis showed that alterations in the chemical compositions of such treated samples occurred with respect to Al, V, and O levels. It was proved that the proliferative capacity of various types of human cells can be modulated by changes in ambient oxygen tension [44].

Carossa et al. [45], in a recent systematic review concerning plasma of argon (PoA), concluded that PoA seems to be effective at promoting cell adhesion and protein adsorption. Tsujita et al. [46] reported in his study that plasma treatment of the implant’s intrabony surface increases the amount of new bone formation in the tissue surrounding the bone. Tsujita has found that the reason for this is atmospheric pressure plasma treatment that improves the wettability of the material surface and reduces ROS [46]. In our study, induction of oxidative stress in cells (ROS) showed a neutral effect of the materials on the level of oxidative stress in the cells. The results of our study suggest that the growth of fibroblasts may be influenced by similar factors to those described for osteoblasts in the case of intraosseous implants with a plasma surface.

## 5. Limitations

A limitation of the study was that a machined titanium sample was not used as a control for cell culture tests. Furthermore, wettability and micro-hardness properties of the modified surfaces should be investigated in the future. Further investigations are required, such as cell colonization, differentiation, and remineralization, to confirm the biological activity.

## 6. Conclusions

Within the limits of the present study, it could be shown that the “S” samples treatment with a low-pressure radiofrequency oxygen plasma had a significant impact on cell growth on the “S”-sample implant surfaces. 

Treatment with low-pressure radiofrequency oxygen plasma significantly enhanced the cytocompatibility of the experimental titanium abutments, and this treatment could be a promising method for clinical application.

## Figures and Tables

**Figure 7:**
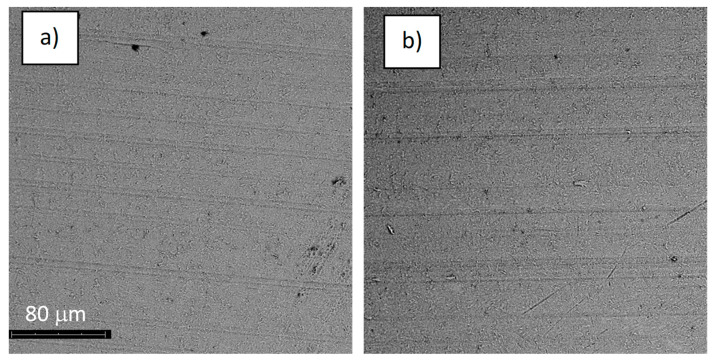
The structure of the surface of the A-28 sample: (**a**) before the corrosion test; (**b**) after the corrosion test; magnification: ×1000.

**Figure 8:**
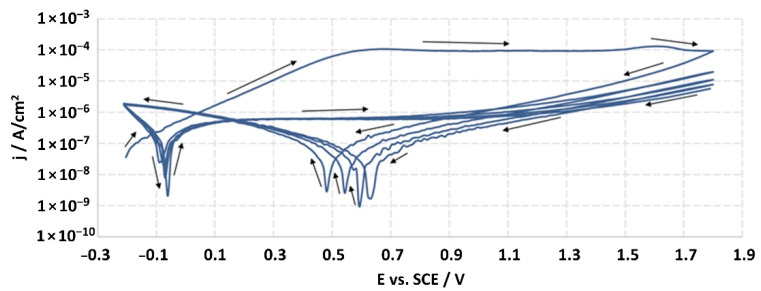
Potentiodynamic curve obtained as a result of cyclic polarization of sample A-28.

**Figure 9:**
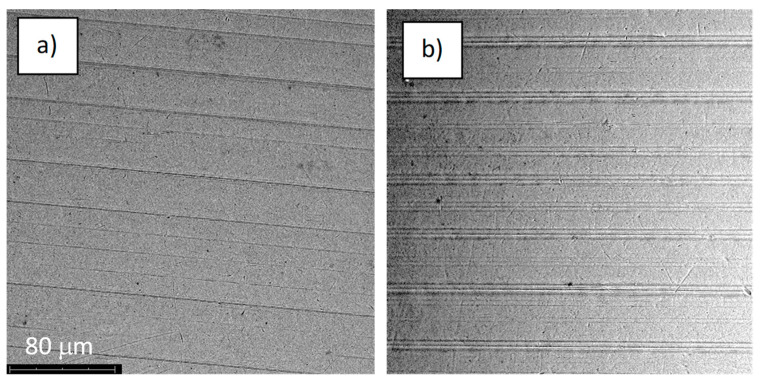
The structure of the surface of the S-28 sample: (**a**) before the corrosion test; (**b**) after the corrosion test; magnification: ×1000.

**Figure 10:**
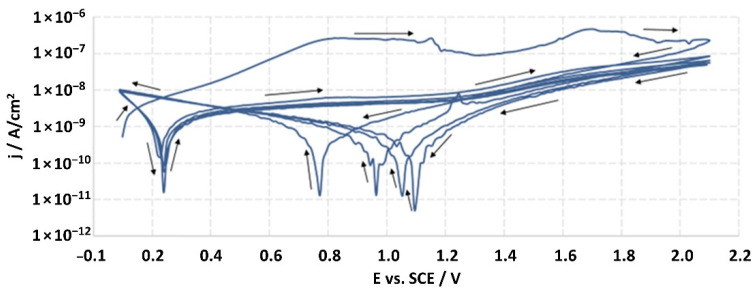
Potentiodynamic curve obtained as a result of cyclic polarization of sample S-28.

**Figure 11:**
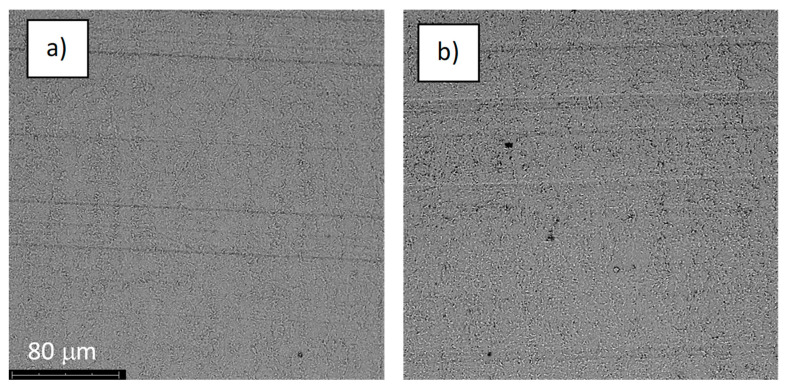
The structure of the surface of sample A-67: (**a**) before the corrosion test; (**b**) after the corrosion test; magnification: ×1000.

**Figure 12:**
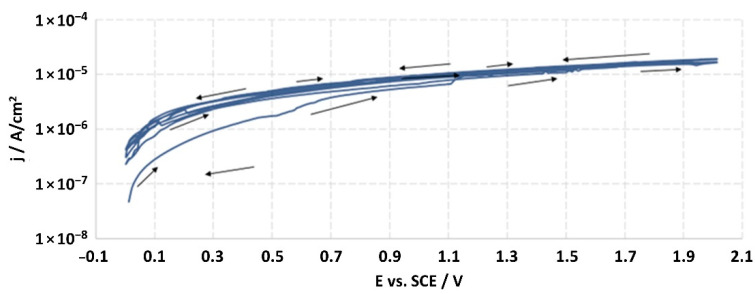
Potentiodynamic curve obtained as a result of cyclic polarization of sample A-67.

**Figure 13:**
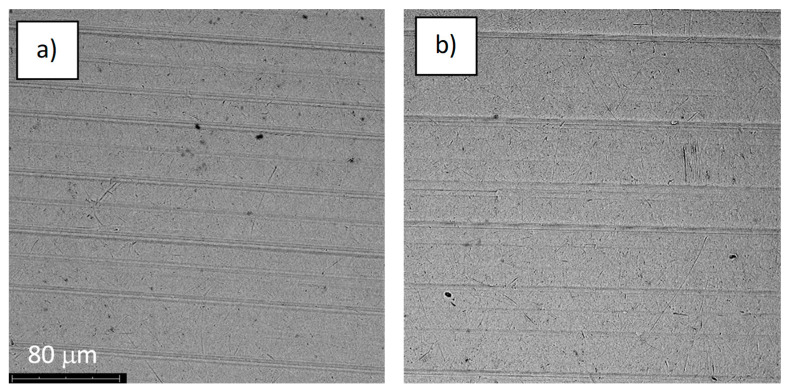
The structure of the surface of sample S-67: (**a**) before the corrosion test; (**b**) after the corrosion test; magnification: ×1000.

**Figure 14:**
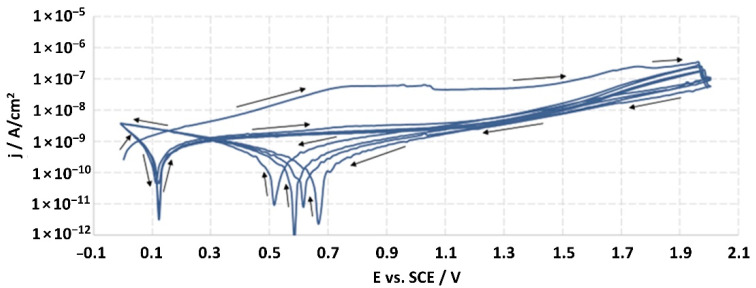
Potentiodynamic curve obtained as a result of cyclic polarization of sample S-67.

**Figure 15:**
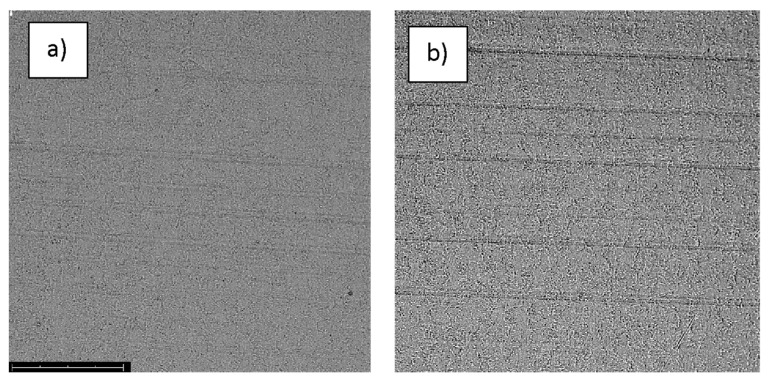
The structure of the surface of sample A-78: (**a**) before the corrosion test; (**b**) after the corrosion test; magnification: ×1000.

**Figure 16:**
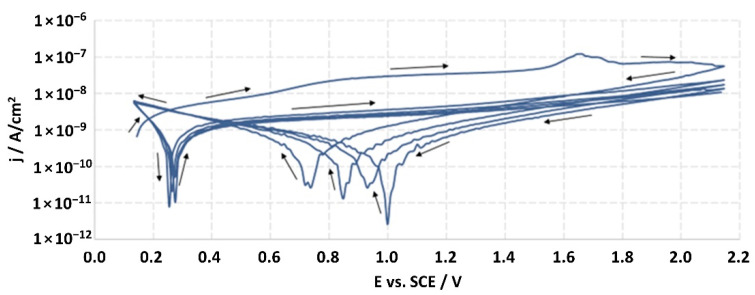
Potentiodynamic curve obtained as a result of cyclic polarization of sample A-78.

**Figure 17:**
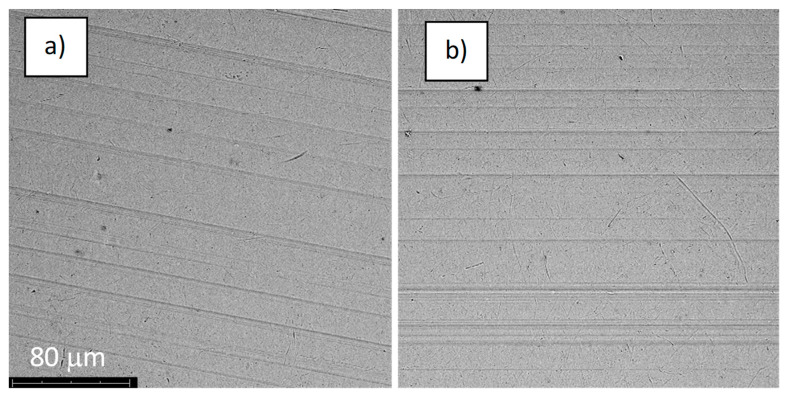
The structure of the surface of sample S-78: (**a**) before the corrosion test; (**b**) after the corrosion test; magnification: ×1000.

**Figure 18:**
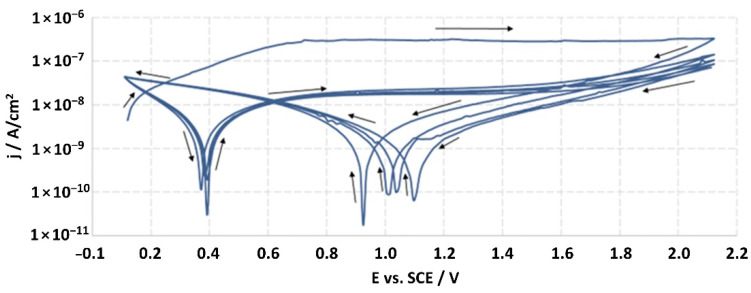
Potentiodynamic curve obtained as a result of cyclic polarization of sample S-78.

**Figure 19:**
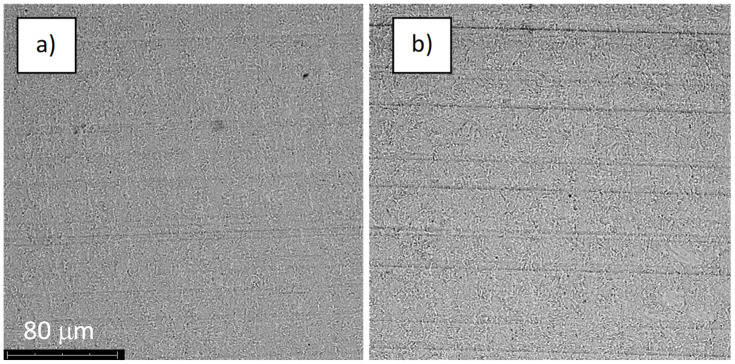
The structure of the surface of sample A-98: (**a**) before the corrosion test; (**b**) after the corrosion test; magnification: ×1000.

**Figure 20:**
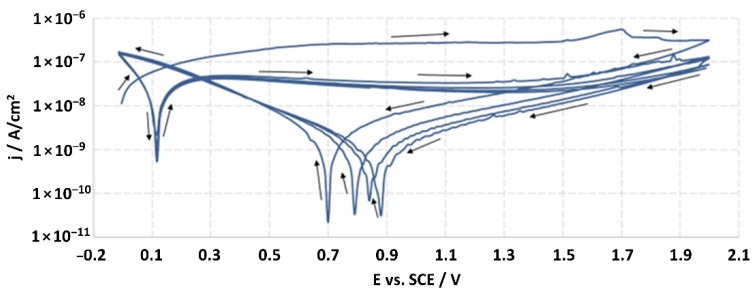
Potentiodynamic curve obtained as a result of cyclic polarization of sample A-98.

**Figure 21:**
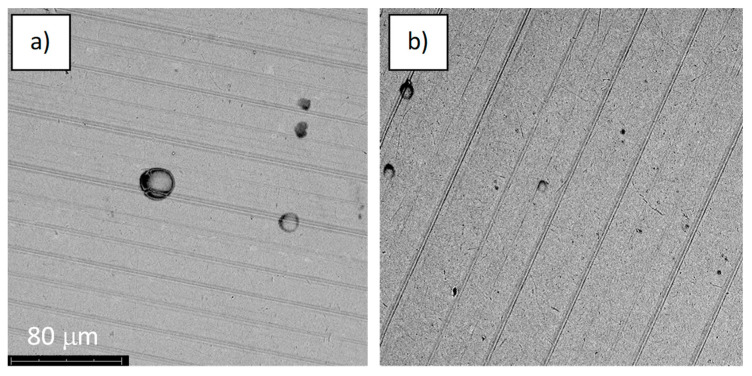
The structure of the surface of sample S-98: (**a**) before the corrosion test; (**b**) after the corrosion test; magnification: ×1000.

**Figure 22:**
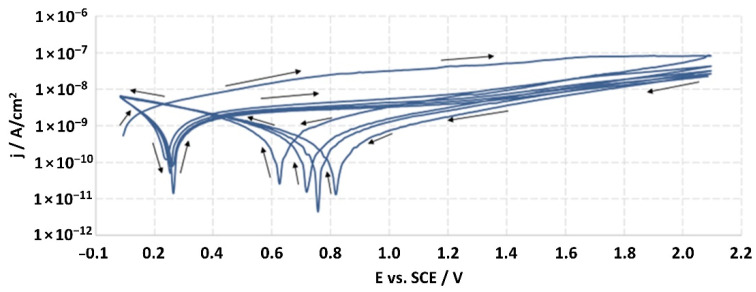
Potentiodynamic curve obtained as a result of cyclic polarization of sample S-98.

**Table 1:** Labeling of samples and anodizing parameters; time—30 s.

Sample No	Electrolyte	Anodizing Voltage, V	Low Pressure RF OP	Sample Color
A-28	H_3_PO_4_	28	+	Blue
S-28				
A-67		67	+	Gold
S-67				
A-78	Na_2_SiO_3_, NaOH, NH_4_F	78	+	Pink
S-78				
A-98		98	+	Green
S-98				

**Table 2:** Semi-quantitative EDX analysis of anodized samples, at %.

	Ti	Al	V	O *	P	Si	F
A-28	37	5	2	56	+		
S-28	38	4	1	57	+		
A-67	33	4	1	62	+		
S-67	32	4	1	63	+		
A-78	32	4	1	63		+	
S-78	31	4	1	64		+	
A-98	32	4	2	62		+	+
S-98	32	3	1	64		+	+

* The values must be considered only informative; “+”—elements were detected with a concentration below 0.3 at %.

## Data Availability

Data available on request.
